# The Analysis of Food and Drugs

**Published:** 1898-06

**Authors:** 


**The Analysis of Food and Drugs.** Part I.—*Milk and Milk Products.* By T. H. Pearmain and C. G. Moor. Pp. viii., 132. London: Baillière, Tindall and Cox. 1897.

This is the first instalment of a promised laboratory handbook, intended to deal with such investigations as constitute the duties of the public analyst: and the wisdom of choosing milk to commence with is obvious when it is remembered that probably no form of sophistication practised at the present moment strikes so directly at the wellbeing of the nation as the adulteration of milk. For not only do the abstraction of cream and the addition of water seriously diminish the food value of milk, but the latter fraud has frequently been the means of introducing specific infections. The authors are sanguine as to the beneficial results of the establishment of legal standards of composition, and would be justified if, as they appear to anticipate, such standards were fixed at or about the average composition of normal milk. It is much to be feared that the opposition would be so strong that any standards legalised would only confirm the present unsatisfactory position.

The legal expert, too, who may consult this work for data upon which to base standards, will find it difficult to form a definite conclusion as to what average milk is. Thus in reference to the proportion of fat in milk, we are told on p. 4 that the average is 3.6 per cent.; on page 11 it is 4 per cent., and that only on the rarest occasions does milk contain less than 3.5 per cent.; whilst on p. 25 the average is given as 3.5 per cent. The highest figure given is probably not far from the truth, seeing that the enormous body of evidence (120,540 samples) collected by Dr. Vieth, and the lengthy researches by the Government chemists quoted on pp. 13–16, agree in recording an average of 4.1 per cent. of butter fat.

The descriptions of processes are clearly worded, and in many cases quoted verbatim from the original: the vexed question of the addition of antiseptics to food is discussed, and methods for their detection in milk, butter, and cheese adequately described. Exception may perhaps be taken to the description of the Reichert process. The method as given is not Reichert's process in its original form, but as improved by Meissl and Wollny; and a very important detail noted by the latter observer is omitted. This is the complete fusion of the fatty acids before distillation is commenced. The neglect of this precaution may result in a reduction of as much as 30 per cent.

in the Reichert figure, and has more than once led to the condemnation of genuine butter in consequence.

Not the least valuable feature of this work is the large number of analyses of foods obtained by modern and trustworthy processes: amongst these may be reckoned those included in the section on cheese, where the composition of many well-known varieties is recorded. When completed this volume is likely to take rank as a standard work of reference in matters relating to the analysis of food and drugs.

